# Predicting the future

**DOI:** 10.7554/eLife.91450

**Published:** 2023-09-06

**Authors:** Greg M Walter, Katrina McGuigan

**Affiliations:** 1 School of Biological Sciences, Monash University Melbourne Australia; 2 School of the Environment, University of Queensland Brisbane Australia

**Keywords:** experimental evolution, quantitative genetics, phenotypic evolution, adaptation, G-matrix, locomotion behavior, *C. elegans*

## Abstract

Experiments on worms suggest that a statistical measure called the G matrix can accurately predict how phenotypes will adapt to a novel environment over multiple generations.

**Related research article** Mallard F, Afonso B, Teotónio H. 2023. Selection and the direction of phenotypic evolution. *eLife* **12**:e80993. doi: 10.7554/eLife.80993.

Predicting how an organism will physically change when adapting to a new environment is a fundamental question in evolutionary biology (Svensson et al., 2021; Walsh and Blows, 2009). However, this is no easy challenge, as changes to one trait may alter another, resulting in unexpected phenotypic outcomes.

One of the main statistical tools scientists use to predict phenotypic evolution is the additive genetic covariance matrix, commonly known as the G matrix. This captures all the genetic variation underlying a set of traits and reveals how this variation influences each of the studied characteristics (Lande, 1979; Walsh and Blows, 2009): for instance, genetic variants that increase the size of individuals may also lead to higher values in other traits, such as speed. Statistical analyses of this matrix can then reveal which combination of trait values has the greatest amount of genetic variation, referred to as ***g***_max_. The genetic variation of a population defines the rate of evolution: the more individuals differ genetically, the faster evolution will occur. Consequently, ***g***_max_ indicates the direction in which a population will evolve the most rapidly. How well ***g***_max_ aligns with the direction of selection (i.e. the set of traits which will impart the highest fitness) then provides a framework for predicting how a population is likely to phenotypically adapt (Figure 1).

**Figure 1.**
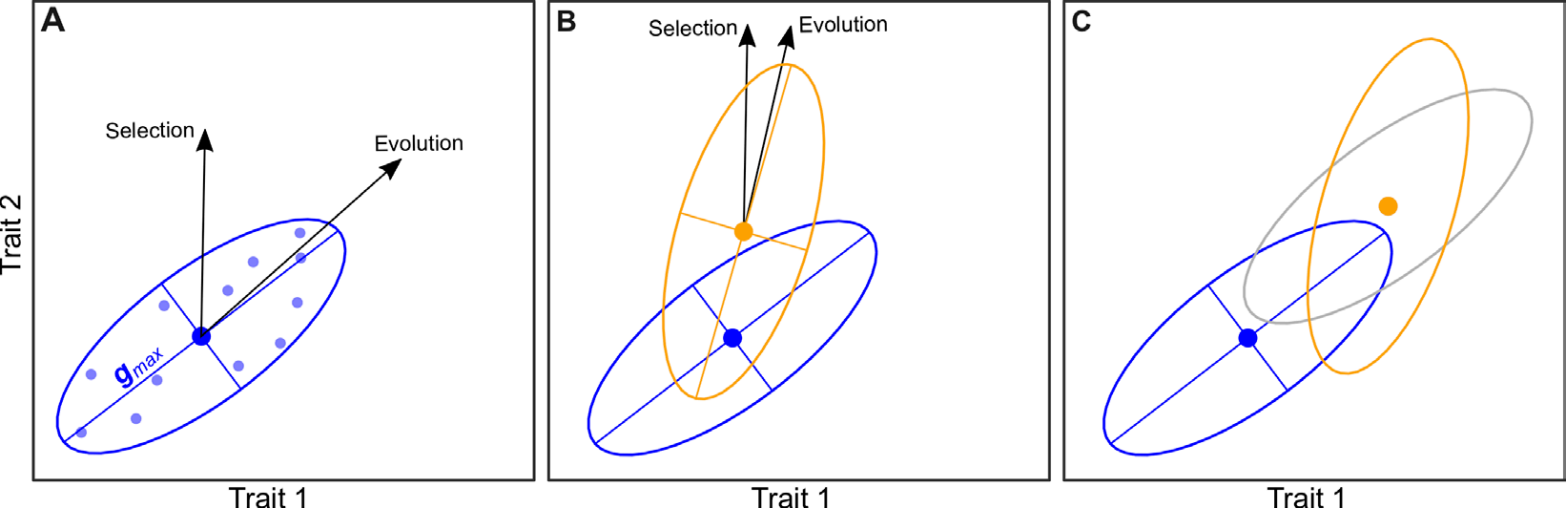
How the genetic variation of a population is distributed among different traits can determine the rate and direction of evolution. (**A**) Mallard et al. studied seven traits in populations of *C. elegans* over 50 generations; one trait was related to body size and six were related to movement. Here, for simplicity, we consider just two traits, and this plot shows that individual organisms (small blue circles) with a high value for trait 1 (horizontal axis) tend to have a high value for trait 2 (vertical axis). We would expect evolution to occur in the direction with the most genetic variance: this direction, which is known as g_max_, is shown by the black arrow labelled evolution. Note that this direction is different from the direction of selection, which is the direction that will result in the largest increase in fitness. The large blue circle shows the mean values of the two traits in the population. (**B**) When a population encounters a new environment, such as a high salt environment in the work of Mallard et al., the values of the traits may change, which could result in a new distribution (orange ellipse), new mean values (large orange circle), and a new direction of evolution. If the direction of evolution rotates towards the direction of selection (as shown here), the rate of adaption will increase, and it if rotates away, the rate of adaption will decrease. (**C**) After several generations of a population adapting to its new environment, the mean values of the traits will have increased (large orange circle), while the distribution of values around the means may remain unchanged (grey ellipse). Alternatively, the distributions might also have evolved (orange ellipse), so the direction of evolution (not shown) will also change.

Observational and manipulative experiments have shown that the G matrix corresponds with how natural populations adapt to different environments (Costa E Silva et al., 2020; Walter, 2023). Indeed, a meta-analysis demonstrated that genetic variation can predict roughly 40% of phenotypic differences in populations of plants (Opedal et al., 2023). However, there are also examples of contemporary evolution not following the predictions of the G matrix (Pujol et al., 2018).

It is possible that instead of guiding the direction of evolution, the G matrix may in fact just become more aligned with phenotypic evolution during adaptation. There is also considerable evidence to suggest that the effect genes have on traits can change across environments (Wood and Brodie, 2015). This could potentially reduce the accuracy of the evolutionary predictions, which assume that genetic variation remains constant even if the environment of a population changes. Now, in eLife, François Mallard, Bruno Afonso and Henrique Teotónio from PSL University in Paris report a series of experiments that test how good the G matrix is at predicting future phenotypes (Mallard et al., 2023a).

Mallard et al. studied the worms *Caenorhabditis elegans* as they were experimentally adapted to environments containing increasingly more salt. First, the team compared worms living in either low or high levels of salt to determine if the genetic variation of the population differed between these two environments. The G matrix of the worms – which encompassed seven traits (one related to body size and the other six to movement) – was similar in both conditions. This suggests that the genetic variation of this initial, ancestral population can predict what will happen to the worms as they gradually adapt to saltier surroundings.

To test this, Mallard et al. adapted three large replicates of the ancestral population (containing over 1,000 worms) to increasing salt concentrations over 35 generations, and then kept them in high salt for a further 15 generations. The worms were then tested to make sure each replicate had evolved higher fitness than the ancestral strain. Mallard et al. found that the mean values of the traits studied (movement and size) evolved in a similar direction to the changes predicted by the G-matrix of their ancestors.

Typically, the G matrix of a populations’ ancestors is unknown. But *C. elegans* can be cryopreserved, meaning Mallard et al. were able to resurrect worms from the ancestral population and measure their G matrix alongside the G matrices of the three evolved groups. This revealed that adaptation to high salt caused the genetic variance of ***g***_max_ to shrink. However, the combination of traits with the most genetic variance did not change (unlike in Figure 1C), suggesting that although selection removed genetic variation as adaptation occurred, the phenotypic evolution of the worms remained predictable.

This study provides strong evidence that the G matrix can retain its predictive ability over evolutionary relevant timeframes (in this case for at least 50 generations). However, major questions about this statistical tool still remain. For instance, can ***g***_max_ ever become aligned with the direction of selection? Does the emergence of new mutations in the genome change the structure of this matrix? Indeed, an earlier study by Mallard and colleagues found that if a mutation was not countered by selection, the set of traits with the most genetic variance would change. This suggests that genetic variation lost because of selection might not be readily replenished by mutations, leading to evolution taking a different direction (Mallard et al., 2023b).

The finding by Mallard et al. that genetic variation is not influenced by the external surroundings of a population is also at odds with previous reports showing genetic effects to depend on the environment (Wood and Brodie, 2015). Further studies experimentally evolving animals in a laboratory may help to resolve how environmental sensitivity of the G matrix influences predictions, as well as provide further insights into the role G matrix plays in predicting evolution.
